# D-Dimers and MPO Are No Suitable Biomarkers for Application in Abdominal Aortic Aneurysm (AAA) Surveillance in a Real-World Setting of Vascular Surgery Patients

**DOI:** 10.3390/biom14121525

**Published:** 2024-11-28

**Authors:** Hans Siegrist, Anja Spieler, Andreas S. Peters, Karola H. Passek, Dittmar Böckler, Susanne Dihlmann

**Affiliations:** 1 Klinik für Gefäßchirurgie und Endovaskuläre Chirurgie, Universitätsklinikum Heidelberg, Im Neuenheimer Feld 420, 69120 Heidelberg, Germany; hans.siegrist@med.uni-heidelberg.de (H.S.); anja.spieler@med.uni-heidelberg.de (A.S.); andreas.peters1@med.uni-heidelberg.de (A.S.P.); passek@med.uni-frankfurt.de (K.H.P.); dittmar.boeckler@med.uni-heidelberg.de (D.B.); 2 Vaskuläre Biomaterialbank Heidelberg (VBBH), Im Neuenheimer Feld 420, 69120 Heidelberg, Germany

**Keywords:** abdominal aortic aneurysm, biomarker, D-dimer, Myeloperoxidase (MPO)

## Abstract

There is currently no clinically valid biomarker for predicting the growth and prognosis of abdominal aortic aneurysms (AAA). The most promising candidates with the highest diagnostic values are plasma D-dimers and markers of activated neutrophils, i.e., myeloperoxidase (MPO) or cell-free DNA. So far, case-control studies on these markers have been performed almost exclusively using healthy individuals as controls. To validate the value of these markers in the clinical setting of a vascular surgery department, we analysed the diagnostic and prognostic potential of plasma D-dimers and MPO in 177 AAA patients versus 138 non-AAA patients with different vascular diseases. Significantly elevated levels of D-dimers were recorded for AAA patients compared with non-AAA patients, although the difference between the two groups was significantly smaller than that in other studies comparing AAA patients with healthy controls. Surprisingly, MPO levels were significantly higher in non-AAA patients than in those with AAA. After adjusting for the confounding factors of sex, peripheral artery disease (PAD) and internal carotid stenosis in multivariate regression models, neither D-dimers nor MPO remained independent correlates of AAA. In contrast, D-dimer plasma levels correlated well with the maximal aortic diameter. Combined analysis of D-dimers and circulating cell-free DNA levels derived from a previous study failed to improve the predictive values for the maximal aortic diameter. In conclusion, our data show that D-dimers and MPO are not suitable biomarkers for monitoring AAA in a real-world setting of mixed vascular surgery patients.

## 1. Introduction

AAA is a chronic disorder characterised by weakening and progressive dilatation of the aorta with an increasing risk of rupture [1,2]. Identification of AAA by screening or incidental imaging at an early stage allows for close surveillance and surgical intervention when the diameter reaches a threshold of 55 mm in male or 50 mm in female patients [3]. However, not all aneurysms expand at the same rate and exhibit the same physical evolution. Therefore, the diameter cannot always predict the rupture risk, which is why the additional use of a specific circulating biomarker would be of great clinical benefit [4]. The pathophysiology of AAA is associated with inflammation, formation of a large intra-luminal thrombus (ILT), and remodelling of the aortic wall. It thus offers numerous approaches for the use of circulating proteins as biomarkers for the prediction of the growth or prognosis of AAA [5,6,7]. Among the most studied and promoted candidates are D-dimers, which were found to have a significant increase in incidence in patients with AAA compared with healthy individuals and were associated with aneurysm growth [7,8,9,10]. D-dimers are cross-linked, fibrin-specific, degradation products, and elevation of D-dimer levels is a sensitive marker for thrombosis [11] and coagulation in clinical practice.

The ILT, which develops in 70–80% of AAA cases [12], may not only release D-dimers but also be a source of other circulating proteins that might be used as biomarkers. Particularly, the accumulation of neutrophils in the ILT and their central role in creating an oxidative and proteolytic environment offers possibilities for monitoring the growth of AAA using neutrophil-related factors [13,14,15]. One of the most abundant proteins in neutrophils, Myeloperoxidase (MPO), which is released when neutrophils are stimulated, has been demonstrated to be associated with AAA development and growth [6,14]. Multivariate regression models using myeloid activation markers and routine laboratory parameters have identified MPO and D-dimer as strong independent correlates of AAA in a study with 41 AAA patients and 38 controls recruited from general surgery, urology and ophthalmology departments [14]. A combined MPO/D-dimer Score recently showed improved performance in distinguishing AAA from peripheral artery disease (PAD) [16]. Moreover, MPO had the best prognostic value, and higher baseline levels of MPO were associated with faster AAA growth in a study population of 65-year-old men invited for an AAA screening programme [6].

Biomarkers for AAA can add additional benefits to the diagnosis and treatment of this disease. Ideally, they are more cost-effective and applicable on a broader patient scale than previous diagnostic tools. Biomarkers could also help in predicting the progression of AAA. The aortic diameter is the most important but not the only predictor of rupture or dissection, and some smaller aneurysms rupture, while larger aneurysms stay constant over a longer period of time. Additional biomarkers could result in better stratification of patients with regard to monitoring and treatment options (conservative vs. operative) [17]. Finally, biomarkers that play a role in the pathogenesis of AAA could serve as possible targets for new medical treatments. Until now, the diagnostic and prognostic values of most circulating biomarkers (including D-dimers and MPO) have been determined in comparison with healthy controls, with groups selected for age and sex (screening programmes), or selected for specific vascular diseases (such as PAD) [8,10,14,16,18,19]. However, a robust biomarker should provide reliable results under routine hospital conditions and be reasonably insensitive to pre-analytical procedures. The aim of our study, therefore, was to validate the diagnostic and prognostic values of D-dimers and MPO for AAA in a clinical real-world setting by using a mixed vascular surgery cohort for analysis. Because anticoagulant tube additives may affect the results of biomarker quantification, we additionally compared the plasma levels of D-dimers and MPO according to the additive that was used during blood collection.

## 2. Materials and Methods

### 2.1. Blood Samples and Pre-Analytics

The study was conducted according to the Code of Ethics of the World Medical Association (Declaration of Helsinki). Patients undergoing treatment at the Klinik für Gefäßchirurgie und endovaskuläre Chirurgie Heidelberg were enrolled in the Vascular Biobank Heidelberg (VBBH) between January 2014 and January 2024. Patient demographics were recorded using the Hospital Information System. The maximum aortic diameter of the AAA patients was determined by computed tomography angiography scan (AAA patients) or ultrasound examination (other vascular patients). Peripheral venous blood was collected as part of a routine set of diagnostic tests on the day of their hospitalisation into lithium-heparin (Sarstedt, Nümbrecht, Germany, S-Monovette, lithium-heparin-Gel) or potassium-EDTA (Sarstedt, Nümbrecht, Germany, S-Monovette, EDTA KE) containing tubes, according to the standard operating procedures of the VBBH. All participants provided written informed consent for the study, which was approved by the ethical committee of the University of Heidelberg (S-025/2023) and (S-310/2013 “Etablierung einer humanen vaskulären Biomaterialbank (VBBH) an der Klinik für Gefäßchirurgie und endovaskuläre Chirurgie der Universitätsklinik Heidelberg), and amendments. Blood was processed for further studies within 4 h of venipuncture. Plasma was collected by centrifugation at 1200× *g* for 15 min. and aliquoted for storage at −80 °C by the VBBH until analysis.

### 2.2. Diagnostic Study Design

Initially, 315 plasma samples were provided by the VBBH for analysis and included according to the following criteria: donors should be over 18 years old and provide informed consent to the study. Exclusion criteria were recent (<1 year) tumour and/or chemotherapy and systemic autoimmune or inflammatory disease. The complete case group consisted of 177 samples from patients who underwent early diagnosis or treatment for repair of their abdominal aortic aneurysm (AAA defined as dilation of all three layers of the aorta; max. diameter ≥ 30 mm; penetrating aortic ulcer (PAU) and dissections of the abdominal aorta were not included), the control group consisted of 138 samples from patients without previous or present AAA (controls, defined by aortic max. diameter < 30 mm) who underwent treatment for other vascular diseases, such as PAD, thrombendarterectomy of the internal carotid artery (ICA), other thrombendarterectomies, varicose veins, minor vascular interventions, and other aortic diseases). When using the lithium-heparin plasma samples only, the groups consisted of 143 AAA and 89 control samples. As not all variables were measurable in all samples, the group sizes on which the calculations were based are explained in the figure legends. Patient characteristics are summarised in Table 1 and Supplementary Table S1.

### 2.3. Analysis of D-Dimers and Myeloperoxidase (MPO) Concentrations in Plasma Samples

Concentrations of D-dimers and MPO in plasma samples were measured by ELISA (Human D-dimer ELISA Kit, Human Myeloperoxidase instant Kit; Thermo Fisher Scientific; Dreieich, Germany) by following the instructions of the manufacturer. Samples were diluted 1:500,000 for D-dimer analysis and 1:50 for MPO analysis. Signal intensity (absorbance) was measured by a TECAN multiplate reader at 450 nm. A reference of 620 nm was used to read the MPO signal intensities.

### 2.4. Statistical Analysis

The sample size of the diagnostic study was calculated based on previously published data on concentrations of D-dimers and MPO in blood plasma [6,14,19,20]. With 150 cases, a power of 83% to show a sensitivity and specificity of at least 0.6 for the marker D-dimers should be achieved. The evaluation on which the power calculation is based tests the union of the two null hypotheses “H1, Sens: Sensitivity ≤ 0.6” and “H1, Spec: Specificity ≤ 0.6” at a one-sided significance level of 2.5%. As this is an intersection union test, the significance level does not need to be adjusted for multiple tests. The concentration of D-dimers was assumed to be log-normally distributed, with medians (IQR) reported in the literature of 1.3 µg/mL (1.68 µg/mL) for AAA patients and 0.47 µg/mL (0.49 µg/mL) for patients without AAA [14,20]. Microsoft Excel was used for data processing. GraphPad Prism 10 software was used for plot preparation, descriptive statistics, and statistical analyses. Data distribution was tested for normality using the Kolmogorov-Smirnov test, Shapiro–Wilk test, or D’Agostino and Pearson test, and appropriate parametric or non-parametric tests were used for further analysis. Multiple group comparisons were carried out using a Kruskal-Wallis test ANOVA or ordinary one-way ANOVA to analyse non-parametric and parametric data, respectively. The box plots show the minimum and the maximum with all data points. The AUC was calculated using the ROC to further characterise the biomarker potential and obtain information on specificity and sensitivity. All specific tests used and *p*-value ranges are indicated in the figure legends. Univariate and multivariate analyses were performed using logistic regression models. *p*-Values for the regression coefficients were calculated using Wald tests. Variables that showed statistical significance with *p*-values < 0.0001 in the univariate analysis were included in the multivariate analysis. The odds ratio (OR) was reported with 95% CI.

## 3. Results

### 3.1. Pre-Analytic Parameters (Anticoagulant, Storage Time) Affecting the Measurements

**Figure 1:**
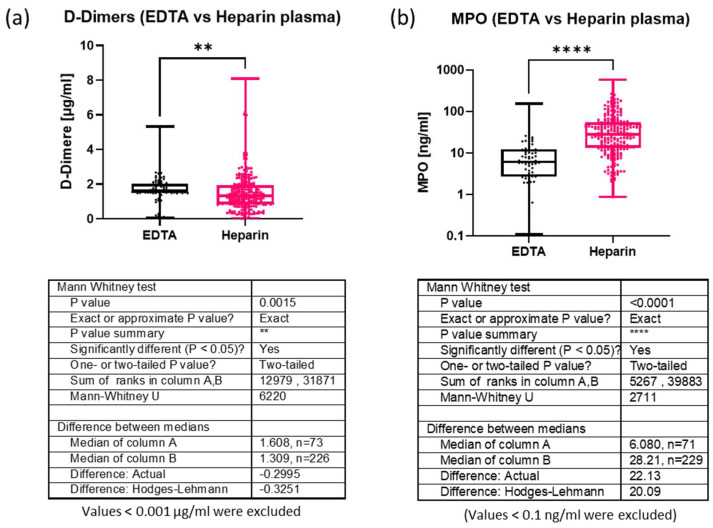
Influence of anticoagulants (EDTA or Heparin) used during blood sampling on the measured plasma levels of D-dimers (**a**) and MPO (**b**). Graphs of boxplots show the interquartile range, medians and maximal and minimal values. Sample sizes, medians and *p*-values for group comparison (based on Mann-Whitney tests) are given in the tables below the boxplots. **: *p* < 0.01, ****: *p* < 0.0001.

Because the plasma samples had been provided from a biobank where they had been collected with different anticoagulants and stored for different time periods, we first determined whether these pre-analytic parameters affected the D-dimer and MPO measurements. The anticoagulant that was used in blood sampling tubes appeared to affect the concentrations of both D-dimers and MPO. The median overall D-dimer concentration was significantly higher in EDTA samples (1.608 µg/mL) than in heparin samples (1.309 µg/mL), with a difference of 0.2995 µg/mL (Figure 1a). In contrast, the median MPO concentration was significantly lower in EDTA samples (6.08 ng/mL) than in heparin samples (28.21 ng/mL), with a difference of 22.12 ng/mL (Figure 1b). Statistical analysis by the Kruskal-Wallis test revealed no significant difference in D-dimer and MPO concentrations between heparin samples that were stored for different lengths of time, although there was a trend for a minimal loss of D-dimers over eight years (Supplementary Figure S1). We, therefore, decided to use only heparin samples for further analysis.

### 3.2. Diagnostic Value of D-Dimers

**Figure 2:**
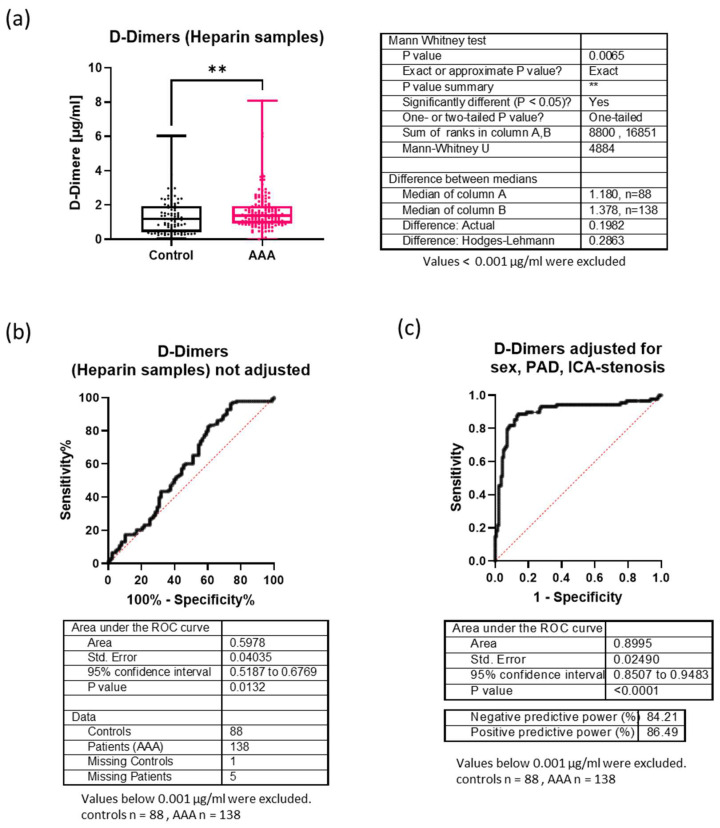
Comparison of D-dimer plasma levels (heparin samples) between AAA patients and controls. (**a**) Boxplots show the interquartile range, medians and maximal and minimal values. Sample sizes, medians and *p*-values for group comparison (based on Mann-Whitney tests) are given in the table next to the graph. **: *p* < 0.01 (**b**) ROC analysis of D-dimer plasma levels in AAA patients against the non-AAA patients (controls) without adjustment. The area under the curve, sample size, 95% confidence interval and *p*-value are given in the table below the graphs. (**c**) ROC analysis of D-dimer plasma levels in AAA patients against the non-AAA patients (controls) after adjustment for sex, PAD and stenosis of the internal carotid artery (ICA). The area under the curve, sample size 95% confidence interval, *p*-value, negative predictive value, and positive predictive value are shown in the table below the graphs.

The heparin plasma levels of D-dimers were significantly increased in AAA patients (median 1.38 µg/mL) compared to controls (median 1.18 µg/mL), *p* = 0.0065, resulting in a small difference of only 0.2 µg/mL between the medians (Figure 2a). Consequently, ROC analysis revealed a low AUC of 0.598 (Figure 2b). A diagnostic logistic regression model was developed that included the following parameters: sex, ICA stenosis, PAD, diabetes, COPD, and previous stroke. After backward elimination, only sex (odds ratio 2.61, 95% CI [1.02–6.72]), ICA stenosis (odds ratio 45.29, 95% CI [18.34–128.3]), and PAD (odds ratio 8.08 and CI [3.27–20.78]) had a significant independent importance for AAA diagnosis, whereas D-dimers had no significant independent importance for AAA diagnosis (odds ratio 0.93 and CI [0.6092–1.343], *p =* 0.737) (Supplementary Table S2). Accordingly, the ROC analysis for D-dimers increased to an AUC of 0.8995 after adjusting for sex, ICA stenosis and PAD. In summary, in our study, D-dimers were not suitable for discriminating patients with AAA from those with other vascular diseases.

### 3.3. Diagnostic Value of Myeloperoxidase (MPO)

**Figure 3:**
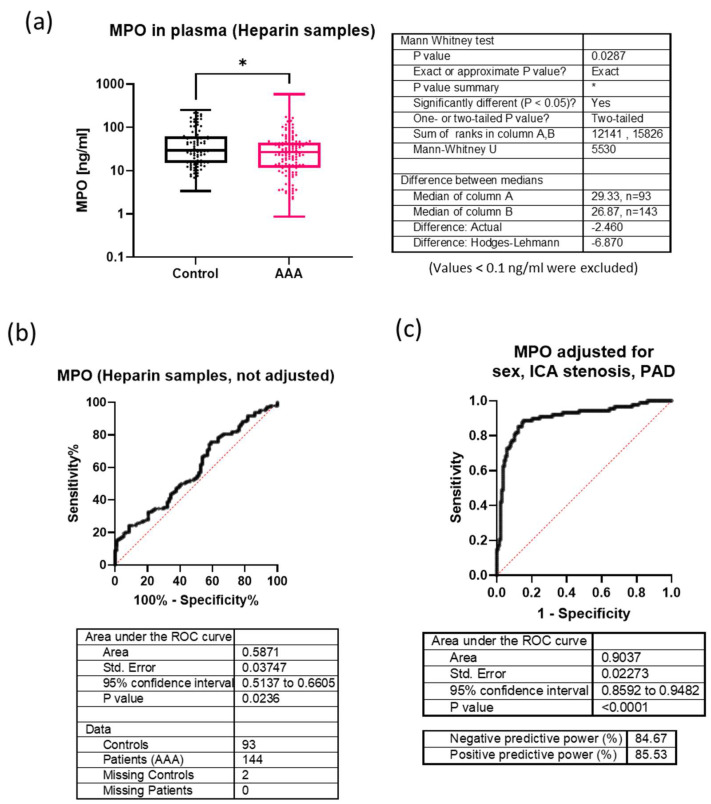
Comparison of MPO plasma levels (heparin samples) between AAA patients and controls. (**a**) Boxplots show the interquartile range, medians and maximal and minimal values. Sample sizes, medians, and *p*-values for group comparison (based on Mann-Whitney tests) are given in the table next to the graph. *: *p* < 0.05 (**b**) ROC analysis of MPO plasma levels in AAA patients compared to non-AAA patients (controls) without adjustment. The area under the curve, sample size, 95% confidence interval, and *p*-value are given in the table below the graphs. (**c**) ROC analysis of MPO plasma levels in AAA patients against the non-AAA patients (controls) after adjustment for sex, PAD and stenosis of the internal carotid artery (ICA). The area under the curve, sample size 95% confidence interval, *p*-value, negative predictive value and positive predictive value are given in the table below the graphs.

The heparin plasma levels of MPO were significantly higher in non-AAA patients (median 29.3 ng/mL) than in AAA patients (median 26.9 ng/mL), *p* = 0.0287, resulting in a small difference of 2.4 ng/mL between the medians (Figure 3a). Consequently, ROC analysis revealed a low AUC of 0.587 (Figure 3b). The diagnostic logistic regression model used for D-dimers was also applied to adjust MPO. After backward elimination, only sex (odds ratio 2.598 and 95% CI [1.01–6.71]), ICA stenosis (odds ratio 46.43 and 95% CI [18.95–130.3]), and PAD (odds ratio 8.09 and CI [3.28–20.78]) had significant independent importance for AAA diagnosis, whereas MPO had no significant independent importance for AAA diagnosis (odds ratio 1.0001 and CI [0.99–1.01], *p =* 0.638) (Supplementary Table S2). Accordingly, the ROC analysis for MPO increased to an AUC of 0.90 after adjusting for sex, ICA stenosis, and PAD. In summary, in our study, MPO was not suitable for discriminating AAA patients from those with other vascular diseases.

### 3.4. Correlation of Plasma Factors with the Maximal Aortic Diameter and Prognostic Values of D-Dimers and MPO for AAA Progression

**Figure 4:**
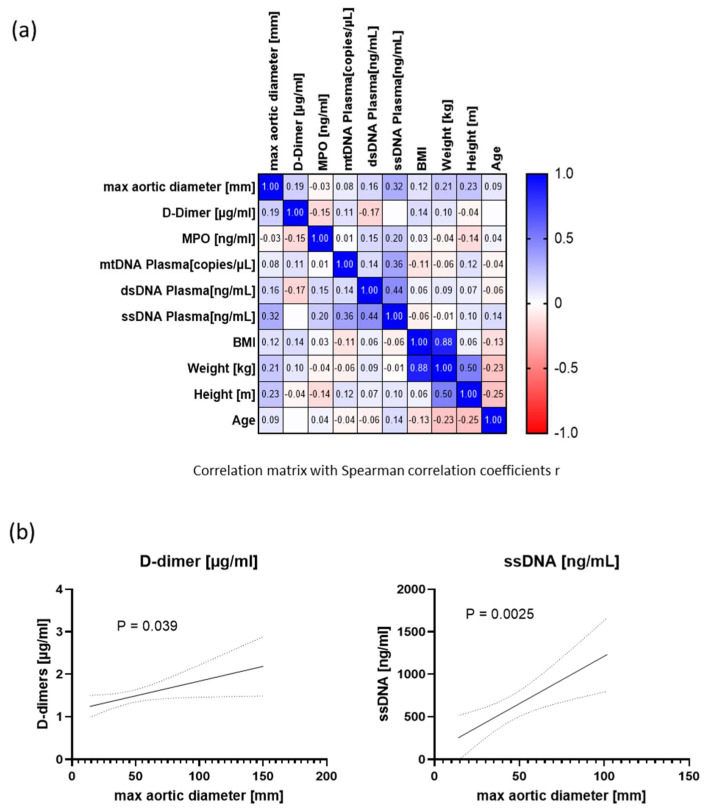
Correlation of different circulating plasma parameters and patient demographics with the maximal aortic diameter. (**a**) Correlation matrix of the age, weight, height, BMI, plasma levels of ssDNA (single-stranded DNA), dsDNA (double-stranded DNA), mtDNA (mitochondrial DNA) copy number, MPO and D-dimers with the maximal aortic diameter of patients with and without AAA. Shown are the Spearman correlation coefficients for each pair of parameters. (**b**) Graphs illustrating simple linear regression models for D-dimers plasma levels (**left panel**) or ssDNA plasma levels (**right panel**) and the maximal aortic diameter of patients with and without AAA.

A subset of the samples used in this study had already been used in a previous study to investigate the diagnostic value of cell-free DNA for monitoring AAA Dihlmann et al. [21]. To determine the correlation across plasma levels of D-dimers, MPO and cell-free DNA with the maximal aortic diameter, a Spearman *r* correlation matrix was performed (Figure 4a and Supplementary Tables S4 and S5). The strongest correlation with maximal aortic diameter was found with cell-free single-stranded (ss) DNA (r = 0.32, *p* = 0.0006). The correlation with D-dimer concentrations was r = 0.19, *p* = 0.0024. The correlation between the maximum aortic diameter and MPO was not significant (r = −0.035, *p* = 0.3041). Of note, the height and weight of the patients also correlated well with the maximum aortic diameter (r = 0.23, *p* < 0.0001 and r = 0.21, *p* = 0.0010, respectively).

To model the prognostic values of the different plasma biomarkers, a simple linear regression analysis was performed, demonstrating an association of the max. aortic diameter with D-dimers (*p* = 0.039) and plasma ssDNA (*p* = 0.0025) (Figure 4b). Plasma levels of MPO, dsDNA and mtDNA revealed no significant association with the max. aortic diameter in simple regression analysis, *p* = 0.956, 0.138, and 0.408, respectively). Finally, using D-dimers and ssDNA in combination did not improve the prognostic value of predicting AAA growth (R-squared in a multiple linear regression model using the two parameters: 0.083).

## 4. Discussion

The association between D-dimer plasma levels and AAA has been well documented [7,8,9,10]. Moreover, a combination of plasma D-dimers and MPO was recently suggested for use in a diagnostic and prognostic AAA score [14] and to distinguish AAA from PAD [16]. Here, we aimed to investigate the diagnostic and prognostic utility of D-dimers and MPO for monitoring AAA in a real-world setting of a typical vascular surgery patient cohort at a university hospital. Accordingly, we compared a group of patients who visited the hospital for surgical treatment or outpatient control of an AAA with a control group of patients with different vascular diseases (non-AAA group).

Because ultrasound screening and CT scans are already well-established non-invasive methods for the diagnostic detection and surveillance of an AAA, additional circulating biomarkers used for diagnostic and prognostic use should be as specific as possible and thus stand out not only from healthy individuals but also from patients with other vascular diseases, such as PAD, carotid stenosis, varicose veins, or others. According to our study results, the difference in the median plasma D-dimer levels between patients with AAA and the control group of non-AAA patients was very low and not suitable for additional diagnostic purposes. With a median D-dimer level of 1.38 µg/mL, our AAA group displayed very similar results to those previously published (0.8 µg/mL [8], 1.30 µg/mL [14,20], 1.27 µg/mL [16], >1.006 µg/mL [10]. In contrast, the median D-dimer level in our non-AAA group (1.18 µg/mL) was much higher than that reported for healthy individuals (0.47 µg/mL [14], 0.38 µg/mL [16], <0.5 µg/mL [10]) or patients with PAD only (0.58 µg/mL [16], 0.28 [8]). Consequently, the area under the ROC curve was very low in our study, resulting in a sensitivity of 60.14% and a weak specificity of 43.06%. At 80% sensitivity, the specificity decreased to less than 40%. For comparison, the cut-off value for D-dimers to rule out thromboembolism is <0.5 µg/mL (fibrinogen equivalent units) and for patients older than 50 years, an adjusted cut-off value (in ng/mL) of age ×10 is recommended [22].

Considering our own results shown here and studies by others [23], we are aware that the choice of anticoagulant supplement (EDTA, heparin, citrate, etc.) during blood sampling affects the measurement of D-dimers. Here, the median D-dimer levels in EDTA plasma samples were significantly higher than those in heparin plasma samples, although both groups contained similar amounts of samples from AAA and non-AAA patients. However, this does not explain the smaller differences between the AAA and control groups using heparin samples only. After adjusting for sex, PAD and ICA stenosis, D-dimer plasma levels were no longer independent markers for AAA prediction in the study presented here, in contrast to sex, PAD and ICA stenosis, which remained independent. This indicates that a great proportion of the D-dimers found in plasma resulted from diseases other than AAA. In line with previous data [8,16], subgroup analysis of our study cohort revealed a trend for increased D-dimer levels in patients with AAA plus PAD compared with patients with PAD only (without AAA), which was not statistically significant (Supplemental Figure S2a). In contrast, accompanying AAA in patients with ICA stenosis did not increase the median D-dimer levels, and the number of patients with AAA and ICA stenosis was very small (Supplemental Figure S2b). In line with our findings, high levels of circulating D-dimer have been previously described in many disorders, and the diagnostic value of D-dimer tests must therefore always be considered in context [24]. For example, increased D-dimer formation has been described in advanced age, recent surgery or trauma, cancer, pregnancy, infection, chronic inflammation, liver disease, renal disease, acute aortic dissection, coronary heart disease and in symptomatic patients undergoing carotid endarterectomy (ICA stenosis) [24,25]. Standardisation of D-dimer assays and further assessment of cut-offs might increase the effectiveness of D-dimer testing, but it remains of limited value when ruling out an AAA.

The second marker that was tested here showed even weaker results. The plasma MPO levels of non-AAA patients were higher than those of patients with AAA, which clearly calls into question the suitability of MPO as a biomarker for AAA. MPO is a lysosomal protein stored in the azurophilic granules of neutrophils, which can be released into the extracellular space during degranulation. The release of MPO is part of the neutrophil defence mechanism to kill pathogens that cannot be phagocytosed, but it can also be detrimental to the host by inducing tissue damage and exacerbating inflammation [26]. Elevated plasma MPO levels are frequently detected in patients with atherosclerosis, cardiovascular, respiratory, autoimmune and neurogenerative diseases and cancer, and correlate with disease severity [26,27,28,29,30]. Thus, considering that the control group here included patients with different vascular diseases, including those resulting from atherosclerosis, the high plasma MPO levels were not surprising. It should be noted here again that the association of increased MPO plasma levels with aortic aneurysm, which has been documented in several studies, resulted from using healthy donors for comparison [6,14,16,31]. In contrast, one study found that decreased serum MPO levels were significantly associated with the risk of peripheral atherosclerosis disease and AAA compared with those in healthy donors [32]. In general, the measured values of our study group were significantly higher than those published earlier (median in AAA (here): 26,87 ng/mL vs. 13.3 ng/mL in AAA and 7.7 ng/mL in healthy donors [14]; 3.01 ng/mL in AAA and 2.79 ng/mL in non-AAA donors from a screening study [6]). This could be due to the different anticoagulants used for blood collection, which resulted in lower readings of samples drawn with EDTA blood collection tubes. In this study, the median plasma MPO levels in samples from EDTA tubes were less than a quarter of those from heparin tubes. Regardless, even when we compared a homogeneous group with samples from heparin tubes only, we could not demonstrate an association between plasma MPO levels and aortic diameter or the occurrence of AAA. Finally, the combination of ssDNA content in plasma examined in a previous study also improved neither the diagnostic nor the prognostic value of D-dimer or MPO levels in plasma.

We are aware that our study has some limitations. First, the use of alternative anticoagulants other than EDTA or heparin, such as citrate, might lead to different measured values. Second, using fresh samples instead of the cryopreserved ones used in this study might give more reliable results, although we did not observe a significant loss of biomarker levels here. In general, identification of prognostic biomarkers for AAA growth will remain challenging, considering that most AAA patients suffer from additional disorders such as PAD, coronary heart disease and ICA stenosis or cancer. Each of these co-morbidities might influence marker levels, which is why any test must be carefully assessed in the context of these co-morbidities. Moreover, for the identification of a prognostic biomarker that might be used for predicting growth or even rupture risk, a correlation of the maximal aneurysm diameter with biomarker levels from different individuals, as it was found for D-dimer levels, is not sufficient. Instead, possible biomarkers need to be measured in the same individual over a longer time period at regular intervals and should correlate with the outcome of interest. Unfortunately, the prediction of rupture, the ultimate goal of biomarker testing, is the most difficult, as no data can be collected due to the need for preventive repair of the AAA. Nevertheless, D-dimer plasma levels might be used for monitoring AAA progression in addition to imaging technologies, such as ultrasound examination or computed tomography (CT) scan, in patients where other D-dimer producing co-morbidities have been excluded. Combining D-dimer levels with one or stronger biomarkers could open new diagnostic windows, especially in targeting aneurysms with small or borderline diameters but with a high risk of rupture based on biomarker levels.

## 5. Conclusions

In summary, we conclude that neither D-dimer nor MPO plasma levels nor a combination of both or cell-free DNA plasma levels are suitable for use as biomarkers for the monitoring and/or prognostics of AAA in the real-world setting of a vascular surgery department. Further research is needed to identify better and more specific biomarkers for AAA. One approach to finding new biomarkers is to search for molecules involved in the pathogenesis and progression of AAA. Another approach could be a multiomics analysis of patients with AAA and the identification of suitable biomarkers with the help of machine learning. No matter which approach will be successful in the future, biomarkers will not replace the conventional imaging modalities in the diagnosis of AAAs. At most, they could add additional biological information, such as wall destruction and inflammatory activity, to the images, thus resulting in better identification of high-risk AAA patients and more personalised therapeutic pathways.

## Figures and Tables

**Table 1:** Patient and control demographics and co-morbidities.

Characteristic	AAA (N = 177) Median (Range)	Non-AAA (N = 138) Median (Range)	*p*-Value
age (years)	71.00 (46–89)	70.00 (29–85)	0.9377
Body mass index (kg/m^2^)	27.29 (17.67–41.03)	25.95 (19.14–42.97)	0.0342
**max. aortic diameter (mm) ^#^**	**58.00 (30–150)**	**19.00 (14–93)**	**<0.0001**
	**N (%)**	**N (%)**	
**Sex**			
** female**	**25 (13.97%)**	**49 (36.03%)**	**<0.0001**
** male**	**154 (86.03%)**	**87 (63.97%)**	
Smoker status			
current + ever	137 (76.54%)	83 (61.48%)	0.0024
never	39 (21.87%)	52 (38.52%)	
unknown	3 (1.68%)	1 (0.74%)	
Hypertension	155 (86.59%)	121 (89.63%)	0.486
Antihypertensive therapy	149 (83.24%)	110 (81.48%)	0.7646
Hypertriglyceridemia *	58 (34.73%)	42 (34.15%)	>0.9999
Hypercholesterinaemia **	160 (95.81%)	122 (99.19%)	0.1442
Lipid-lowering therapy	145 (81.01%)	113 (83.70%)	0.5555
**ICA stenosis**	**8 (4.60%)**	**83 (63.36%)**	**<0.0001**
**PAD**	**21 (11.73%)**	**59 (43.70%)**	**<0.0001**
Antiplatelet therapy	137 (76.54%)	110 (81.48%)	0.3311
Coronary heart disease	96 (53.63%)	58 (42.96%)	0.0685
Myocardial infarction	40 (22.35%)	18 (13.33%)	0.0556
Stroke	15 (8.38%)	26 (19.26%)	0.0064
Diabetes	31 (17.32%)	42 (31.11%)	0.0047
Metformin therapy	21 (11.73%)	23 (17.04%)	0.1922
COPD	21 (11.73%)	8 (5.93%)	0.1140
Chronic kidney disease	27 (15.08%)	27 (18.52%)	0.4457
AAA family history	12	0	
no family history	18		
unknown	149		

AAA: abdominal aortic aneurysm; ICA; internal carotid artery; PAD: peripheral artery disease; COPD: chronic obstructive pulmonary disease; IQR: Interquartile range; ^#^ available for all AAA and 109 non-AAA; non-AAA includes patients with dilative aorta due to PAU (n = 4) or dissections of the thoracic aorta (n = 4); * TAG > 150 mg/dL; ** total cholesterol > 100 mg/dL.

## Data Availability

All data required for interpretation can be found in the manuscript and Supplementary Materials. The underlying raw data for the measurements will be provided by the corresponding author upon reasonable request.

## Supplementary Materials

The following supporting information can be downloaded at https://www.mdpi.com/article/10.3390/biom14121525/s1; Supplemental Figure S1: Effects of the storage time on the median plasma concentration of D-dimers and MPO; Supplemental Figure S2: Comparison of D-dimers plasma levels (Heparin samples) between subgroups (excluding patients with PAU or thoracic aortic dissection); Supplemental Table S1: Patient and control blood parameters; Supplemental Table S2: Multiple logistic regression of D-dimers, adjusted for sex, ICA stenosis, PAD (Heparin plasma only); Supplemental Table S3: Multiple logistic regression of MPO, adjusted for sex, ICA stenosis, PAD MPO (Heparin plasma only); Supplemental Table S4: Correlation of maximal aortic diameter with continuous variables (Heparin plasma only). Supplemental Table S5: One-tailed *p*-values of Spearman correlation, maximal aortic diameter with other variables (Heparin plasma only).

## References

[1] Davis F.M., Daugherty A., Lu H.S. (2019). Updates of Recent Aortic Aneurysm Research. Arter. Thromb. Vasc. Biol..

[2] Golledge J., Moxon J.V., Singh T.P., Bown M.J., Mani K., Wanhainen A. (2020). Lack of an effective drug therapy for abdominal aortic aneurysm. J. Intern. Med..

[3] Moll F.L., Powell J.T., Fraedrich G., Verzini F., Haulon S., Waltham M., van Herwaarden J.A., Holt P.J., van Keulen J.W., Rantner B. (2011). Management of abdominal aortic aneurysms clinical practice guidelines of the European society for vascular surgery. Eur. J. Vasc. Endovasc. Surg. Off. J. Eur. Soc. Vasc. Surg..

[4] Kurvers H., Veith F.J., Lipsitz E.C., Ohki T., Gargiulo N.J., Cayne N.S., Suggs W.D., Timaran C.H., Kwon G.Y., Rhee S.J. (2004). Discontinuous, staccato growth of abdominal aortic aneurysms. J. Am. Coll. Surg..

[5] Groeneveld M.E., Meekel J.P., Rubinstein S.M., Merkestein L.R., Tangelder G.J., Wisselink W., Truijers M., Yeung K.K. (2018). Systematic Review of Circulating, Biomechanical, and Genetic Markers for the Prediction of Abdominal Aortic Aneurysm Growth and Rupture. J. Am. Heart Assoc..

[6] Memon A.A., Zarrouk M., Agren-Witteschus S., Sundquist J., Gottsater A., Sundquist K. (2020). Identification of novel diagnostic and prognostic biomarkers for abdominal aortic aneurysm. Eur. J. Prev. Cardiol..

[7] Nana P., Dakis K., Brodis A., Spanos K., Kouvelos G. (2021). Circulating Biomarkers for the Prediction of Abdominal Aortic Aneurysm Growth. J. Clin. Med..

[8] Cai H., Pan B., Xu J., Liu S., Wang L., Wu K., Yang P., Huang J., Wang W. (2022). D-Dimer Is a Diagnostic Biomarker of Abdominal Aortic Aneurysm in Patients With Peripheral Artery Disease. Front. Cardiovasc. Med..

[9] Golledge J., Muller R., Clancy P., McCann M., Norman P.E. (2011). Evaluation of the diagnostic and prognostic value of plasma D-dimer for abdominal aortic aneurysm. Eur. Heart J..

[10] Sundermann A.C., Saum K., Conrad K.A., Russell H.M., Edwards T.L., Mani K., Bjorck M., Wanhainen A., Owens A.P. (2018). Prognostic value of D-dimer and markers of coagulation for stratification of abdominal aortic aneurysm growth. Blood Adv..

[11] Ariens R.A., de Lange M., Snieder H., Boothby M., Spector T.D., Grant P.J. (2002). Activation markers of coagulation and fibrinolysis in twins: Heritability of the prethrombotic state. Lancet.

[12] Harter L.P., Gross B.H., Callen P.W., Barth R.A. (1982). Ultrasonic evaluation of abdominal aortic thrombus. J. Ultrasound Med..

[13] Piechota-Polanczyk A., Jozkowicz A., Nowak W., Eilenberg W., Neumayer C., Malinski T., Huk I., Brostjan C. (2015). The Abdominal Aortic Aneurysm and Intraluminal Thrombus: Current Concepts of Development and Treatment. Front. Cardiovasc. Med..

[14] Zagrapan B., Eilenberg W., Prausmueller S., Nawrozi P., Muench K., Hetzer S., Elleder V., Rajic R., Juster F., Martelanz L. (2019). A Novel Diagnostic and Prognostic Score for Abdominal Aortic Aneurysms Based on D-Dimer and a Comprehensive Analysis of Myeloid Cell Parameters. Thromb. Haemost..

[15] Klopf J., Brostjan C., Neumayer C., Eilenberg W. (2021). Neutrophils as Regulators and Biomarkers of Cardiovascular Inflammation in the Context of Abdominal Aortic Aneurysms. Biomedicines.

[16] Zagrapan B., Klopf J., Celem N.D., Brandau A., Rossi P., Gordeeva Y., Szewczyk A.R., Liu L., Ahmadi-Fazel D., Najarnia S. (2023). Diagnostic Utility of a Combined MPO/D-Dimer Score to Distinguish Abdominal Aortic Aneurysm from Peripheral Artery Disease. J. Clin. Med..

[17] Golledge J., Tsao P.S., Dalman R.L., Norman P.E. (2008). Circulating markers of abdominal aortic aneurysm presence and progression. Circulation.

[18] Folsom A.R., Yao L., Alonso A., Lutsey P.L., Missov E., Lederle F.A., Ballantyne C.M., Tang W. (2015). Circulating Biomarkers and Abdominal Aortic Aneurysm Incidence: The Atherosclerosis Risk in Communities (ARIC) Study. Circulation.

[19] Vele E., Kurtcehajic A., Zerem E., Maskovic J., Alibegovic E., Hujdurovic A. (2016). Plasma D-dimer as a predictor of the progression of abdominal aortic aneurysm. J. Thromb. Haemost..

[20] Eilenberg W., Zagrapan B., Bleichert S., Ibrahim N., Knobl V., Brandau A., Martelanz L., Grasl M.T., Hayden H., Nawrozi P. (2021). Histone citrullination as a novel biomarker and target to inhibit progression of abdominal aortic aneurysms. Transl. Res..

[21] Dihlmann S., Kaduk C., Passek K.H., Spieler A., Böckler D., Peters A.P. (2024). Exploring circulating cell-free DNA as a biomarker and as an inducer of AIM2-inflammasome-mediated inflammation in patients with abdominal aortic aneurysm. Sci. Rep..

[22] Righini M., Van Es J., Den Exter P.L., Roy P.M., Verschuren F., Ghuysen A., Rutschmann O.T., Sanchez O., Jaffrelot M., Trinh-Duc A. (2014). Age-adjusted D-dimer cutoff levels to rule out pulmonary embolism: The ADJUST-PE study. JAMA.

[23] Favresse J., Lippi G., Roy P.M., Chatelain B., Jacqmin H., Ten Cate H., Mullier F. (2018). D-dimer: Preanalytical, analytical, postanalytical variables, and clinical applications. Crit. Rev. Clin. Lab. Sci..

[24] Weitz J.I., Fredenburgh J.C., Eikelboom J.W. (2017). A Test in Context: D-Dimer. J. Am. Coll. Cardiol..

[25] Krupinski J., Catena E., Miguel M., Domenech P., Vila R., Morchon S., Rubio F., Cairols M., Slevin M., Badimon L. (2007). D-dimer local expression is increased in symptomatic patients undergoing carotid endarterectomy. Int. J. Cardiol..

[26] Rizo-Tellez S.A., Sekheri M., Filep J.G. (2022). Myeloperoxidase: Regulation of Neutrophil Function and Target for Therapy. Antioxidants.

[27] Baldus S., Heeschen C., Meinertz T., Zeiher A.M., Eiserich J.P., Munzel T., Simoons M.L., Hamm C.W., Investigators C. (2003). Myeloperoxidase serum levels predict risk in patients with acute coronary syndromes. Circulation.

[28] Brennan M.L., Penn M.S., Van Lente F., Nambi V., Shishehbor M.H., Aviles R.J., Goormastic M., Pepoy M.L., McErlean E.S., Topol E.J. (2003). Prognostic value of myeloperoxidase in patients with chest pain. N. Engl. J. Med..

[29] Nicholls S.J., Hazen S.L. (2005). Myeloperoxidase and cardiovascular disease. Arter. Thromb. Vasc. Biol..

[30] Zhu A., Ge D., Zhang J., Teng Y., Yuan C., Huang M., Adcock I.M., Barnes P.J., Yao X. (2014). Sputum myeloperoxidase in chronic obstructive pulmonary disease. Eur. J. Med. Res..

[31] Brandau A., Ibrahim N., Klopf J., Hayden H., Ozsvar-Kozma M., Afonyushkin T., Bleichert S., Fuchs L., Watzinger V., Nairz V. (2022). Association of Lipoproteins with Neutrophil Extracellular Traps in Patients with Abdominal Aortic Aneurysm. Biomedicines.

[32] Pradhan-Palikhe P., Vikatmaa P., Lajunen T., Palikhe A., Lepantalo M., Tervahartiala T., Salo T., Saikku P., Leinonen M., Pussinen P.J. (2010). Elevated MMP-8 and decreased myeloperoxidase concentrations associate significantly with the risk for peripheral atherosclerosis disease and abdominal aortic aneurysm. Scand. J. Immunol..

