# Supplementary material for: D-Dimers and MPO Are No Suitable Biomarkers for Application in Abdominal Aortic Aneurysm (AAA) Surveillance in a Real-World Setting of Vascular Surgery Patients
Source: Biomolecules. 2024 Nov 28;14(12):1525. doi: 10.3390/biom14121525 (PMC11673383; doi:10.3390/biom14121525)
Supplement: Supplementary file 1 [file biomolecules-14-01525-s001.zip › biomolecules-3297741-supplementary.pdf]

Supplemental materials to

D-dimers and MPO are no suitable biomarkers for application in abdominal aortic aneurysm (AAA) surveillance in a real-world setting of vascular surgery patients

By

Hans Siegrist[1], Anja Spieler[1], Andreas S. Peters[1,2], Karola H. Passek[1,3], Dittmar Böckler[1,2], and Susanne Dihlmann[1*]

1 Universitätsklinikum Heidelberg, Klinik für Gefäßchirurgie und endovaskuläre Chirurgie, Im Neuenheimer Feld 420, 69120 Heidelberg, Germany

2 Vaskuläre Biomaterialbank Heidelberg (VBBH), Im Neuenheimer Feld 420, 69120 Heidelberg, Germany, E-Mail: Andreas.Peters1@med.uni-heidelberg.de

3 present address: Universitätsklinikum Frankfurt, Klinik für Herz- und Gefäßchirurgie, Theodor-Stern-Kai 7, 60596 Frankfurt am Main, Germany, E-Mail: passek@med.uni-frankfurt.de

* Correspondence: e-mail: Susanne.dihlmann@med.uni-heidelberg.de, Tel.: +49-6221-5639908

Supplemental Table S1: **Patient and control blood parameters**

Characteristic	AAA (N = 177)<br>*Median (IQR)*	Non-AAA (N = 138)<br>*Median (IQR)*	*P*-value
**Characteristic**	**AAA (N = 177)**<br>***Median (IQR)***	**Non-AAA (N = 138)**<br>***Median (IQR)***	***P*-value**
White blood cells (cells/nl)	7,61 (2,28)	7,98 (2,96)	0,1513
**Erythrocytes (cells/nl)**	**4,57 (0,72)**	**4,35 (0,69)**	**0,0001**
CRP (mg/l)*	2,4 (5)	2,2 (5,25)	0,9789
Triglycerides (mg/dl)	119 (80,5)	121 (89,55)	0,6547
Total Cholesterol (mg/dl)	154 (51)	159 (60)	0,0600
LDL-Cholesterol (mg/dl)**	77,50 (38,5))	82,50 (47,55)	0,9788
HDL-Cholesterol (mg/dl)**	41 (16)	46,5 (20)	0,1030
Creatinine (mg/dl)	0,99 (0,33)	0,98 (0,48)	0,6491

CRP: C-reactive protein; LDL: low-density lipoprotein; HDL: high-density lipoprotein; eGFR: estimated glomerular filtration rate; *CRP < 2 mg/l was set to 1 mg/l; ** only available for 79 AAA and 54 Non-AAA;

Supplemental Table S2: Multiple logistic regression of D-dimers, adjusted for sex, ICA stenosis, peripheral artery disease (Heparin plasma only)

**Odds ratios**	**Variable**	**Estimate**	**95% CI (profile likelihood)**
**β0**	Intercept	0,1167	0,04898 to 0,2598
**β1**	D-Dimers [ng/µl]	0,9346	0,6092 to 1,343
**β2**	Male sex [0]	2,616	1,023 to 6,727
**β3**	ICA stenosis (surgery indication)	45,29	18,34 to 128,3
**β4**	periphereal artery disease[1]	8,083	3,272 to 20,78
Sig. diff. than zero?	**Variable**	**|Z|**	**P value**
**β0**	Intercept	5,074	<0,0001
**β1**	D-Dimers [ng/µl]	0,3349	0,7377
**β2**	Male sex [0]	2,012	0,0442
**β3**	ICA stenosis (surgery indication)	7,752	<0,0001
**β4**	periphereal artery disease[1]	4,455	<0,0001

0: negative, 1: positive

Supplemental Table S3: Multiple logistic regression of MPO, adjusted for sex, ACI stenosis, peripheral artery disease MPO (Heparin plasma only)

Odds ratios	Variable	Estimate	95% CI (profile likelihood)
**β0**	Intercept	0,09877	0,04957 to 0,1812
**β1**	MPO [ng/µl]	1,001	0,9947 to 1,007
**β2**	Male sex [0]	2,598	1,010 to 6,711
**β3**	ICA stenosis (surgery indication) [1]	46,43	18,95 to 130,3
**β4**	periphereal artery disease[1]	8,090	3,276 to 20,78
**Sig. diff. than zero?**	**Variable**	**|Z|**	**P value**
**β0**	Intercept	7,048	<0,0001
**β1**	MPO [ng/µl]	0,4702	0,6382
**β2**	Male sex [0]	1,987	0,0469
**β3**	ICA stenosis (surgery indication) [1]	7,871	<0,0001
**β4**	periphereal artery disease[1]	4,459	<0,0001

0: negative, 1: positive

Supplemental Table S4: Correlation of maximal aortic diameter with continuous variables (Heparin plasma only)

	Spearman r	P-values (one-tailed)
**Age**	0,091	0,0905
**Height [m]**	0,233	3,3105e-004
**Weight [kg]**	0,212	0,0010
**BMI**	0,116	0,0478
**ssDNA Plasma[ng/mL]**	0,321	0,0006
**dsDNA Plasma[ng/mL]**	0,156	0,0601
**mtDNA Plasma[copies/µL]**	0,077	0,2217
**MPO [ng/ml]**	-0,035	0,3041
**D-Dimer [µg/ml]**	0,190	0,0024

Supplemental Table S5: One-tailed P values of spearman correlation, maximal aortic diameter with other variables (Heparin plasma only)

	Age	Height [m]	Weight [kg]	BMI	ssDNA Plasma [ng/mL]	dsDNA Plasma [ng/mL]	mtDNA Plasma [copies/µL]	MPO [ng/ml]	D-Dimer [µg/ml]	Max. aortic diameter [mm]
**Age**		1,0745e -004	2,1171e -004	0,0263	0,0779	0,2787	0,3311	0,2546	0,4716	0,0905
**Height [m]**	1,0746e -004		1,5357e -015	0,1820	0,1634	0,2392	0,1154	0,0176	0,2785	3,3105e -004
**Weight [kg]**	2,1171e -004	1,5358e -015		1,4901e -071	0,4491	0,1906	0,2697	0,2623	0,0771	0,0010
**BMI**	0,0263	0,1820	1,4901e -071		0,2629	0,2761	0,1402	0,3315	0,0208	0,0477
**ssDNA Plasma [ng/mL]**	0,0779	0,1635	0,4491	0,2629		1,1585e -006	1,0753e -004	0,02	0,4811	0,0006
**dsDNA Plasma [ng/mL]**	0,2787	0,2393	0,1906	0,2761	1,1585e -006		0,0771	0,0643	0,0423	0,0601
**mtDNA Plasma [copies/µL]**	0,3312	0,1155	0,2697	0,1402	1,0753e -004	0,0771		0,4657	0,1220	0,2217
**MPO [ng/ml]**	0,2547	0,0176	0,2623	0,3315	0,02	0,0643	0,4657		0,099	0,3041
**D-Dimer [µg/ml]**	0,4717	0,2785	0,0772	0,0209	0,4811	0,0423	0,1220	0,099		0,0025
**max aortic diameter [mm]**	0,0905	3,3106e -004	0,0010	0,0477	0,0006	0,0600	0,2217	0,3041	0,0025	

Blue: P values < 0.01

Supplemental Figure S1, Siegrist et al., 2024

(a)

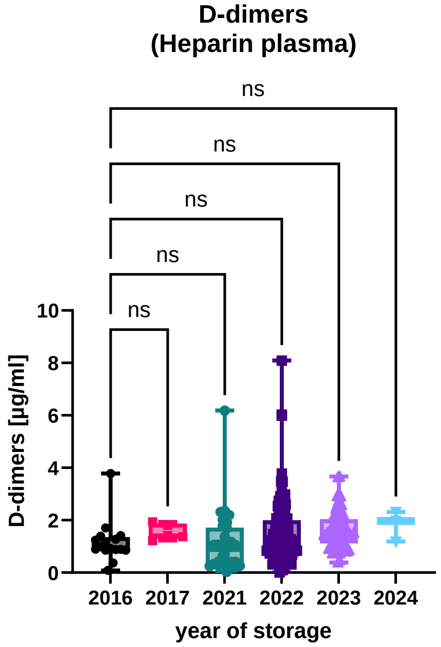

Test for linear trend	
Slope	0.1382
SE of slope	0.06330
95% CI of slope	0.01344 to 0.2629
P value	0.0301
P value summary	*
Is linear trend significant (P < 0.05)?	Yes

(b)

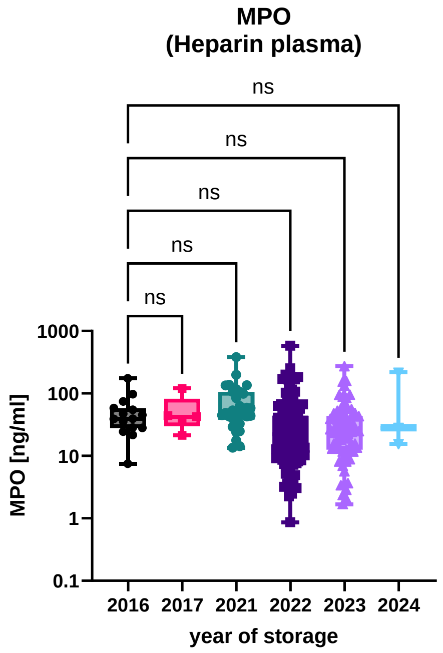

Test for linear trend	
Slope	-6.027
SE of slope	-3.862
95% CI of slope	-13.64 to 1.583
P value	0.1200
P value summary	ns
Is linear trend significant (P < 0.05)?	No

Effects of the storage time on the median plasma concentration of D-dimers (A) and MPO (B). Statistical analysis by Kruskal Wallis test revealed no significant difference between younger and older heparin plasma samples (D-dimers: P = 0.211; MPO: P = 0.085). An ordinary one-way ANOVA test for linear trend revealed a trend towards a slight loss of D-dimer concentrations in older samples (slope = 0.138; P = 0.030), whereas there was no trend towards loss of MPO detectability in older samples (P = 0.57). No Heparin plasma samples were available from 2018-2020.

Supplemental Figure S2, Siegrist et al., 2024

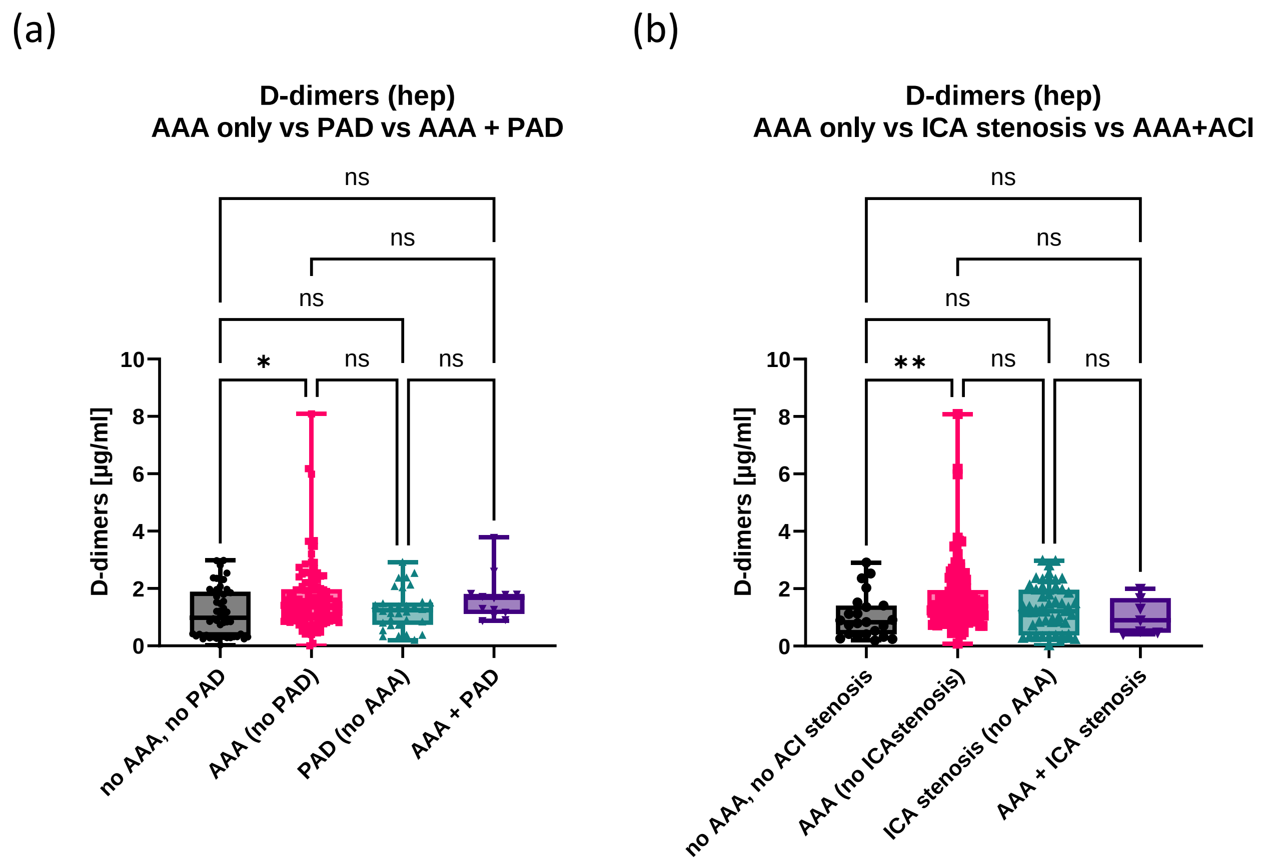

		(a)						(b)			
n	=	50	122	32	13	n	=	23	124	58	7
Median (µg/ml)	=	0,981	1,371	1,235	1,672	Median (µg/ml)	=	0,838	1,398	1,213	0,896

Supplemental Figure S2. Comparison of D-dimers plasma levels (Heparin samples) between subgroups (excluding patients with PAU or thoracic aortic dissection).(a) D-dimer levels were devided into 4 subgroups drived from: I) patients without AAA or PAD versus II) patients with only AAA (without PAD) versus III) patients with PAD (without AAA) IV) patients with AAA plus PAD. (b) D-dimer levels were devided into 4 subgroups drived from: I) patients without AAA or ICA stenosis versus II) patients with only AAA (without ICA stenosis) versus III) patients with ICA stenosis (without AAA) IV) patients with AAA plus ICA stenosis. Data were statistically analysed by one-way ANOVA (Kruskal-Wallis test and Dunn's test multiple comparison). * P < 0.05; ** P < 0.01, ns: not significant.
